# Experiences of persons with multiple sclerosis of a collaborative care programme: A qualitative study

**DOI:** 10.1002/nop2.1893

**Published:** 2023-06-30

**Authors:** Firuzeh Payamani, Seyed Reza Borzou, Alireza Soltanian, Masoud Ghiasian, Mahnaz Khatiban

**Affiliations:** ^1^ Social Determinants of Health Research Center Aligoudarz School of Nursing Lorestan University of Medical Sciences Khorramabad Iran; ^2^ School of Nursing and Midwifery Hamadan University of Medical Sciences Hamadan Iran; ^3^ Chronic Diseases (Home Care) Research Center Department of Medical‐Surgical Nursing School of Nursing and Midwifery Hamadan University of Medical Sciences Hamadan Iran; ^4^ Modeling of Noncommunicable Diseases Research Center Hamadan University of Medical Sciences Hamadan Iran; ^5^ Department of Neurology Medical School Hamadan University of Medical Sciences Hamadan Iran; ^6^ Mother and Child Care Research Center, Department of Ethics Education in Medical Sciences Department of Medical‐Surgical Nursing School of Nursing and Midwifery Hamadan University of Medical Sciences Hamadan Iran

**Keywords:** decision‐making, multiple sclerosis, nurse–patient relationship, patient‐centred care

## Abstract

**Aim:**

This study aimed to explain the experiences of individuals with multiple sclerosis (MS) about the collaborative care programme.

**Design:**

This qualitative study was conducted from July 2021 to March 2022.

**Methods:**

We conducted this study with individuals with MS who participated in the collaborative care programme in Hamadan, Iran. A purposive sampling with maximum variety was applied to recruit patients until data saturation. Eventually, 18 patients consented and were interviewed using a semi‐structured interview guide. The transcriptions of audio‐checked interviews were analysed using a conventional content analysis approach of Graneheim and Lundman by MAXQDA 10, 2010 edition.

**Results:**

The study identified three main categories. that emerged from the participants' experiences of collaborative care: the ‘Beginning of Communication’, which included two subcategories, ‘Introduction and Acquaintance with Each Other’ and ‘Formation of Trust’; ‘Mutual Interaction’, which included three subcategories, ‘Dialogue’, ‘Mutual Goal Setting’ and ‘Mutual Agreement of Care Solutions’; and ‘Exchange of Targeted Behaviors’, which included six categories, Implementation of Strategies for ‘Nutritional Behaviors’, ‘Sleep and Rest’, ‘Constipation Relief’, ‘Promotion of Physical Activity and Exercise’, ‘Fatigue Reduction’ and ‘Stress Management’.

**Conclusions:**

The findings highlight the statistically significant role of collaborative care in MS management. Utilizing these research findings can update the development of interventions based on collaborative care, which can provide appropriate support to individuals with MS.

**Patient or public contribution:**

Individuals with multiple sclerosis.

## 1 INTRODUCTION

Multiple sclerosis (MS) is an autoimmune condition with acute and chronic inflammatory lesions in the central nervous system (CNS), leading to tissue damage and disability of the affected person (Koch‐Henriksen & Magyari, 2021). Currently, 2.8 million people worldwide are diagnosed with MS, and its prevalence has increased in recent years (Kassie et al., 2021; Walton et al., 2020). The MS prevalence in Iran is about 5.3 to 89 per 100,000 people, and its incidence rate is 7 to 1.148 per 100,000 people (Azami et al., 2019). There are 2000 MS patients in Hamadan province, which is high compared with the province population and shows that Hamadan is one of the most prolific provinces in terms of the prevalence of this disease (Ghiasian, 2019).

The disease is associated with the involvement of different parts of the central nervous system. Therefore, individuals' symptoms can vary from benign condition to rapidly progressive and debilitating disease that requires extensive lifestyle adaptation (Harrison & AS, 2004). The problems caused by the disease can affect patients' lives and cause disturbances in activities related to wellness, thereby leading to secondary health consequences, the loss of individual independence and the reduction in the capacity to perform daily living activities (Dehghani et al., 2019).

Factors such as fatigue, receiving medication (Farran et al., 2020), disturbed role performance related to physical health problems (Heidari Sureshjani et al., 2012), and physical restrictions in one or more fields decline the quality of life (QOL) among individuals with MS (Buzaid et al., 2013). Active participation of the patient in the care process in chronic conditions such as MS is associated with positive health‐related outcomes (Payamani et al., 2020), and collaboratively management is intended for this disease (Rahimi‐Bashar et al., 2020).

Collaborative care is a systematic and rational process of establishing practical, interactive and dynamic communication between the client and the healthcare providers. This process leads to a greater understanding and recognition of needs and health issues to control the disease. Accordingly, it motivates and involves the client to accept responsibility and be an active healthcare team member to improve wellness (Mohammadi et al., 2002). Although patient engagement, involvement and participation have semantic differences and similarities, from a nursing perspective, these concepts reflect the process of collaborative care, which involves mutual participation between the patient and nurse. To make this process unique, interactive actions are required from both parties, including asking questions, sharing information, acquiring knowledge, making decisions and teaching for patients, and diagnosing, responding, sharing information, teaching and collaborating for nurses (Jerofke‐Owen et al., 2023).

One of the theories related to collaborative care is King's theory of goal attainment. The central concept of this theory is collaborative care which emphasizes the patient's participation in decisions such as determining and agreeing on self‐care goals, prioritizing goals, agreeing with methods to achieve goals and understanding the patient's direction. In this regard, determining the degree of goal attainment is highly emphasized (Fawcett & Desanto‐Madeya, 2012).

Based on King's theory, nurses can recognize the patient's understanding by establishing interaction and reaching a joint agreement to determine the mutual goals of self‐care, prioritizing goals and methods of achieving goals (King, 2007). By involving patients in goal setting, nurses allow them to make decisions for their health and express their perceptions of illness, related health problems, experiences and tensions (Adib‐Hajbaghery & Tahmouresi, 2018). Creating and fostering a two‐way nurse–patient relationship encourages clients to participate in counselling sessions and express their experiences and feelings (Nasab et al., 2017). This approach is strongly recommended to acknowledge the positive effects of collaborative care in improving the quality of care for chronic diseases (Rahimi‐Bashar et al., 2020). The results of some studies in mutual collaborative care in the management of chronic diseases indicate that this approach is a necessary element to promote the client's trust in healthcare providers and ultimately improve the health outcomes, QoL and satisfaction of the affected (Rahimi‐Bashar et al., 2020; Shamsi et al., 2017), and reduces nurses' workload (Chan et al., 2018).

Although collaborative care has been acknowledged as an effective method for managing chronic disorders, there has been a lack of qualitative research exploring patient experiences and perceptions of collaborative care. The purpose of conducting this study was to fill a gap in the existing literature regarding the perceptions of individuals with MS who receive collaborative care. Understanding the experiences of individuals with MS who receive collaborative care can inform the development and implementation of more patient‐centred care approaches.

## 2 THE STUDY

### 2.1 Design

This qualitative study with a conventional content analysis approach was undertaken after a quantitative study to implement collaborative care for people with multiple sclerosis (MS). The study period was from July 2021 to March 2022.

### 2.2 Sample and setting

The research population consisted of individuals with MS who participated in the collaborative care programme at the Neshat Rehabilitation Center in Hamadan, Iran. Inclusion criteria were an age range of 20–50 years, confirmation of the disease by a neurologist, at least 6 months passed since diagnosis, and suffering from relapsing–remitting MS. To assess patients' orientation to time, person and place, they were asked to repeat their full name, present location and the current date. Purposive sampling with maximum diversity was used to recruit patients until data saturation was achieved. Maximum variation was ensured based on the participants' demographic characteristics and interventional study results. None of the participants were excluded. Sampling continued until data saturation, which occurred after the fifteenth interview. Additionally, three more interviews were conducted to ensure sampling adequacy.

### 2.3 Data collection

The quantitative study results were utilized as a guide for the subsequent qualitative phase, which involved conducting interviews using a conventional content analysis approach. The interviews were designed by research team and conducted by a Nursing Ph.D. candidate, who invited participants either in person or by phone at the Rehabilitation Center. The participants were provided with detailed information about the study and informed consent for audio recording was obtained. To confirm the suitability of the semi‐structured interview guide, the research team assessed the initial interviews. The primary questions of the interview were shown in Table 1.

**TABLE 1:** Interview guide questions with participants.

No	Interview guide questions
1	Please describe your experience of participating in care sessions
2	What did the nurse do in these care sessions?
3	Describe how you participate in these care sessions.
4	Express your experience after participating in care sessions
5	What did you expect participation in care sessions to do for you?

During the interviews, follow‐up and exploratory questions such as ‘Please elaborate on this?’ and ‘What do you mean?’ were used based on participants' responses. The interviews were scheduled in advance and conducted in a private room within the community building. Each participant was interviewed individually in a face‐to‐face setting. The duration of the interviews ranged from 30 to 60 min based on the physical condition and willingness of the participants, with descriptive notes taken to supplement the findings. The theoretical saturation of the categories in the study was reached after the 15th interview, with an additional three interviews conducted to ensure adequate sampling.

### 2.4 Data analysis

The collected data underwent analysis based on Lundman and Graneheim's five‐step procedure (Graneheim & Lundman, 2004). Firstly, the text was transcribed instantly after each interview. Secondly, the interview transcript was reread to gain a general insight. Thirdly, each interview's entire transcript was considered a unit of analysis, with meaningful units specified and coded in the fourth step. Based on the continuous comparison of similarities, differences and appropriateness, codes with a single topic were merged into one subcategory, and then, the subcategories were put into categories. Finally, by comparing the subcategories and categories and conducting deep and detailed reflection, the content embedded in the data was introduced under the title of main classes. The data were analysed in MAXQDA 10, 2010 edition.

### 2.5 Validity and reliability

To ensure the trustworthiness of the research, Guba and Lincoln's proposed criteria were employed (Hsieh & Shannon, 2005). The researchers aimed to enhance credibility by adopting long‐term involvement throughout the research, effectively interacting with the participants to verify the accuracy of the information and coding. Reflexivity was employed to prevent potential biases in the findings. As such, the researcher documented their feelings and experiences in a daily notebook at the beginning and during the research process, enabling them to understand the participants' experiences without influencing their assumptions. The dependability was improved by repeating the steps of data collection and analysis and seeking input from colleagues and experts. Colleague approval and additional comments were used to increase the conformability of the data. To promote transferability, the research report provided a detailed description of the study, enabling evaluation of the research's applicability in other domains.

### 2.6 Ethics

The Hamadan University of Medical Sciences and Health Services approved the study proposal with the number 9910096961. Also, the proposal was approved by the Ethics Committee with the number IR.UMSHA.REC.1399.773. Verbal and written informed consent was obtained from the participants before the study's start and the interviews' recording. Participants were free to voluntarily participate in or withdraw from the study and were assured that the information would be kept confidential. The first researcher allowed participants to call or text them via WhatsApp Messenger if they had questions or needed information.

## 3 RESULTS

The mean age of participants was 36.94 ± 9.27 years and with an average illness duration of 8.08 ± 5.57 years, and other demographic information of the participants is summarized in Table 2.

**TABLE 2:** Demographic characteristics of the participants.

No.	Gender	Age	Marital status	Educational level	Occupation	Illness duration (year)	Interview time (minutes)
1	Female	31	Single	Diploma	Self–employment	16	60
2	Female	22	Single	Bachelor	University student	1	30
3	Female	34	Single	Master	Employee	19	45
4	Female	27	Married	Diploma	Housewife	7.5	30
5	Female	46	Married	High school	Housewife	6	45
6	Female	33	Married	Master	Housewife	13	40
7	Male	44	Married	Bachelor	Retired	15	45
8	Female	49	Married	Bachelor	Employee	2	35
9	Female	41	Married	Bachelor	Housewife	9	48
10	Female	49	Single	Diploma	Self–employment	5	45
11	Female	21	Single	Diploma	Housewife	11	30
12	Female	47	Divorced	Diploma	Housewife	8	42
13	Male	37	Single	Diploma	Unemployed	1	36
14	Female	42	Married	Diploma	Housewife	13	34
15	Male	33	Married	Master	Self–employment	7	30
16	Female	25	Single	Diploma	Housewife	9	35
17	Female	47	Married	High school	Employee	2	30
18	Male	37	Married	High school	Manual worker	1	30

Following analysis of the interview data, 375 initial codes were extracted, which were subsequently merged due to semantic similarities, resulting in 92 codes. The participants experienced collaborative care as a continuous, systematic, and logical process that involved three categories: ‘initiation of communication’, ‘mutual interaction’, and ‘exchange of targeted behaviors’ (Table 3).

**TABLE 3:** Categories and subcategories extracted from codes obtained from the experiences of participants.

Theme	Category	Subcategory
Collaborative care	Initiation of communication	Introduction and acquaintance with each other
		Formation of trust
	Mutual interaction	Dialogue
		Mutual goal setting
		Mutual agreement on care solutions
	Exchange of targeted behaviours	Implementation of strategies for nutritional behaviours
		Implementation of strategies for sleep and rest
		Implementation of strategies for constipation relief
		Implementation of strategies for the promotion of physical activity and exercises
		Implementation of strategies for fatigue reduction
		Implementation of strategies for stress management

### 3.1 Category 1—Initiation of communication

The ‘initiation of communication’ was a process in which the nurse and patient introduced themselves. It comprised two categories: ‘Introduction and Acquaintance with Each Other’ and ‘Formation of Trust’.

#### 3.1.1 1–1 Introduction and acquaintance with each other

This stage aimed to facilitate familiarity between the nurse and client, with the aim of establishing a relationship between them. The participants frequently mentioned this relationship in their conversations. As one participant noted:

‘The first day I saw you were at the Neshat Clinic. You came to me very friendly and introduced yourself. You said you were a student doing research, and you told me you would like to work with me. I said yes, and then you asked your questions. You asked, and I gave your answer with satisfaction and ….’ (p4, 27‐year woman)

#### 3.1.2 1–2 Formation of trust

After the introduction and familiarization process, establishing the client's trust was crucial for their involvement with the collaborative care plan. Many participants articulated confidence in the nurse–patient relationship, without which the patient's participation in the care would have been limited. Participants noted that the nurse's appearance and behaviour were important factors in building trust between them. As one participant stated:

‘I met you on the first day and saw that I could trust you to tell you all my problems. When you talked to me, you gave me peace. I spoke to you, and I found peace.’ (p11, 21‐year woman)

### 3.2 Category 2—Mutual interaction

This class involved a mutual and purposeful interaction between the nurse and the patient. Establishing effective communication and interaction between the two parties facilitated decision‐making and increased the patient's involvement in the care process. This category emerged from of three categories: ‘dialogue’, ‘mutual goal‐setting’ and ‘mutual agreement on care solutions’.

#### 3.2.1 2–1 Dialogue

The nurse engaged in simple and clear discussions with the patient regarding their medication, disease, complications and problems. As one participant noted:

‘The first day I saw you, you talked to me about my illness. You asked how I got sick. What symptoms did I have? I explained ….’ (p16, 25‐year woman)

#### 3.2.2 2–2 Mutual goal setting

The nurse and patient mutually made decisions on care solutions, such as identifying and agreeing on self‐care goals, prioritizing goals, coordinating for achieving goals and understanding the patient. As one participant stated

‘We set goals together, such as improving nutrition, exercising and walking. And you put the goal of improving nutrition as the first preference.’ (p2, 22‐year woman)

#### 3.2.3 2–3 Mutual agreement on care solutions

Based on the research findings, a mutual agreement was reached between the nurse and client regarding realistic, achievable, measurable and evaluable care goals. One participant noted:

‘You explained to me about relieving constipation that I should drink a glass of water every morning without fasting. I can use a variety of laxative foods such as plums, stewed plums, and olives, or eat high‐fiber foods, every day. We planned our life situation.’ (p18, 37‐year man)

### 3.3 Category 3—Exchange of targeted behaviours

The third class extracted from the collaborative care process was the ‘Exchange of Targeted Behaviors’, which involved meaningful interactions between patients and nurses aimed at achieving self‐care goals. Effective interaction occurred when both parties shared similar goals and engaged in purposeful behaviours to achieve them. During the exchange, patients effectively performed their roles. The exchange of targeted behaviours comprised six categories, Implementation of Strategies for ‘"Nutritional Behaviors’, ‘Sleep and Rest’, ‘Constipation Relief’, ‘Promotion of Physical Activity and Exercise’, ‘Fatigue Reduction’ and ‘Stress Management’.

#### 3.3.1 3–1 Implementation of strategies for nutritional behaviours

The experiences of patients with MS indicate the importance of improving their nutritional behaviours in the collaborative care programme. As one contributor stated:

‘I used to consume a lot of salt with food. Now I don't use things that I think are harmful to me. I use olive oil. During the day, I eat olives and almonds, use them every night, and eat dates for my daily food and use more fruits with meals.’ (p6, 33‐year woman)

#### 3.3.2 3–2 Implementation of strategies for sleep and rest

Participants' experiences indicated improved sleep and rest patterns after the collaborative care programme. Most of them had sleep disorders before the intervention, and strategies were developed based on mutual goals between the nurse and patient to improve the quality of sleep. As one participant noted:

‘I used to wake up at 11:00 in the morning and stay awake until 3:00 or 4:00 o'clock. Now, at 11:00 p.m., I turn off my mobile phone. I close the curtains in the room and sleep until 5:00. I wake up on my own again. I wake up at 5 or 6. I sleep for 8 hours a day and do not wake up in between.’ (p1, 31‐year woman)

#### 3.3.3 3–3 Implementation of strategies for constipation relief

Patients with MS often experienced elimination disorders due to inactivity and medication side effects, resulting in the frequent use of laxatives. After the collaborative care programme, participants used different strategies to relief constipation. One participant stated:

‘I was always constipated, but now I drink enough fluids every day. I learned that dried figs are very good for constipation, my bowel movements are much better now, and my stomach works once a day.’ (p7, 44‐year man)

#### 3.3.4 3–4 Implementation of strategies for promotion of physical activity and exercise

Patients also emphasized the importance of physical activity and exercise. As one contributor noted:

‘I used to exercise irregularly. I didn't go for walks anymore. But since you told me, I walk and exercise. I have a stationary bike at home.’ (p4, 27‐year woman)

#### 3.3.5 3–5 Implementation of strategies for fatigue reduction

Fatigue was a common side effect of MS disease and medication. Patients reported fatigue repeatedly, for example: one of the participants said: ‘I always felt tired …’ (p1, 31‐year woman). The participants leaned some alternatives to reduce fatigue, including balancing activity and rest, improving sleep, managing stress and engaging in physical activity. One participant noted:

‘When I was walking, I took a short break, even if I had to sit for two minutes. Now, if I go for a long walk and I rest for 5‐6 minutes. Going to a sports club has made me feel much better in terms of not getting tired.’ (p1, 47‐year woman)

#### 3.3.6 3–6 Implementation of strategies for stress management

Patients also reported being able to manage their stress through the collaborative care programme. One participant noted different stress reduction methods, stating:

‘…I do not bring negative energy into my mind, and I do not think about negative things. Every night, I spend a long time alone, 5 to 10 minutes. I write down a series of things. It was so nice.’ (p7, 44‐year man)

## 4 DISCUSSION

The objective of this study was to explore the experiences of individuals with MS who participated in a collaborative care programme. The findings revealed that the participants perceived collaborative care as three interrelated processes: ‘Initiation of Communication’, ‘Mutual Interaction’ and ‘Targeted Behavior Exchange’.

Our findings on collaborative care align with previous research suggesting that patient participation in decision‐making and treatment implementation can lead to better healthcare services (Dobscha et al., 2009; Ebrahimi et al., 2017) increased self‐sufficiency in self‐care, and improved motivation, responsibility and participation in treatment, ultimately resulting in higher quality care (Alidina et al., 2021; Foroutani et al., 2014; Rakhshan et al., 2018). Partnerships emerged as an essential theme in a qualitative study to explore what individuals with MS want and need to manage their communication changes better. The participants expressed a desire to promote self‐care participation through effective interaction alongside healthcare providers. They suggested routine assessment, access to more information, a comprehensive programme to provide interventions, availability of services, support groups and increased awareness of MS disease as ways to manage their illness better (El‐Wahsh et al., 2022).

### 4.1 The beginning of communication

Effective communication is a critical element in forming a collaborative care relationship between nurses and patients. The beginning of communication is especially important, as it sets the foundation for ongoing interactions. In our study, the classes of emerged as crucial, with two categories: ‘Introduction and Acquaintance with Each Other’ and ‘Formation of Trust’, as stated by most participants. It is a common observation that nurses often struggle to communicate effectively with patients and can only do so through a patient‐centred approach (McCabe, 2004). A descriptive study in Iran revealed that 80% of patients hospitalized in the burn department did not know their nurses, and nurses were only present during medicine administration and dressing. The study also identified a statistically significant correlation between nurse–patient communication and patient satisfaction (Lotfi et al., 2019).

Our study suggests that effective interaction between nurses and patients begins with the nurse's acquaintance with the patient and trust establishment. In different cultures, the nurse–patient connection and trust are the foundation of nursing communication and patient‐centred care (Minton et al., 2022). The introduction process of nurses to patients (Guest, 2016) and trust form the foundations of the therapeutic nurse–patient relationship (Allande‐Cussó et al., 2022). Trust is developed over time during ongoing nurse–patient interactions and can be strengthened or diminished. Once gained, patient trust in the nurse must be maintained, as it is a fragile element that is not easily regained once lost (Rajcan et al., 2020). From the patient's perspective, the nurses' personal and professional characteristics are essential in trust development. Conversely, mistreatment, professional incapability and communication problems are contributors to mistrust (Ozaras & Abaan, 2018). A systematic review of patients' experiences revealed that knowledge and commitment in nurses are associated with promoting trust (Rørtveit et al., 2015). In a review study, it was found that nurses and patients who established trust had better adaptability and cooperation to improve health. They also expressed a sense of security and a desire to participate more confidently (Leslie & Lonneman, 2016). As a result, nurses can initiate a patient‐centred approach by introducing themselves and building trust. Additional researches are needed to further explore the process of ‘Beginning of Communication’. Investigating this process can help to identify effective approaches to building trust and rapport between nurses and patients, ultimately improving the quality of care provided to individuals with chronic illnesses.

### 4.2 Mutual interaction

In our study, mutual interaction involved a shared and purposeful collaboration between the nurse and the patient. Establishing effective nurse–patient communication facilitated the patient's involvement in the decision‐making. Mutual interaction between nurse and patient provides care and improves health outcomes, empowerment and security (Allande‐Cussó et al., 2022). The collaborative approach between the healthcare system, patients and families, and their social system requires mutual and interactive cooperation (Costa et al., 2022). Effective mutual communication between patients and healthcare providers is crucial for patient care and recovery (Kwame & Petrucka, 2021).

Collaborative care involves a continuous process of mutual interaction through nurse–patient ‘dialogue’, ‘mutual goal‐setting’ and ‘mutual agreement on care solutions’. In clinical nursing, many practices such as patient assessment, education and counselling are dialogue‐based (Crawford et al., 2017). Nursing is a live dialogue and an intersubjective exchange where nurses and patients engage in friendly communication to present their experiences (O'Connor, 1992). A respectful open dialogue between the patient and care provider is crucial in recognizing needs, promoting positive care outcomes, and understanding the quality of care (Kwame & Petrucka, 2021). The dialogical approach of nurses can reduce patients' reluctance and open their minds to accept care advice (Wesseldijk‐Elferink et al., 2021). Nurse–patient dialogue can prepare patients with enough information about mutual goal setting and effective participation in the decision‐making process. Nurses' role in providing patient readiness improves the mutual goal‐setting process for patients (Vaalburg et al., 2021).

Providing mutual goal setting based on respect for patients' needs is essential in promoting outcomes and understanding the quality of care. By taking into account patients' preferences and values in joint decision‐making, further improving patients' abilities enhances their satisfaction and agreement with the treatment procedure (Ben‐Zacharia et al., 2018). Patient‐centred care is based on valuing patients' experiences and knowledge, providing care focusing on their values, priorities and needs, and respecting the patient by involving them more in the self‐care process (Johnsson et al., 2018).

Our results support mutual goal setting in the collaborative care process, leading to later joint agreement on care solutions and strategies. Nurses typically write and document a patient care plan based on their professional experience and knowledge, which can be seen as a violation of person‐centred care, as the nurse ignores the patient's resistance and avoids shared status. In collaborative care, care strategies should be developed by the professional and the patient together, with both taking the initiative in the actual writing (Forsgren & Björkman, 2021). However, communication with patient and mutual decision‐making are two critical factors in patients' adherence with treatment. As a result, the stronger the communication and the more patients participate in their treatment decisions, the greater their adherence with treatment will be (Yu et al., 2022). Further studies are needed to explore the concept of mutual interaction, which involves shared and purposeful collaboration between the nurse and the patient.

#### 4.2.1 Exchange of targeted behaviours

The collaborative care process for individuals with MS involves the ‘Exchange of Targeted Behaviors’, which refers to meaningful interactions between patients and nurses aimed at achieving self‐care goals and strategies. Effective interaction occurs when both parties share care goals and strategies and engage in purposeful behaviours to achieve them. During the exchange, patients effectively perform their roles. A consultation care plan in self‐management interventions leads to the realization of outcomes such as self‐efficacy among individuals with MS (Witzig‐Brändli et al., 2023).

Even during the COVID‐19 pandemic, a nurse–patient interaction derived from King's theory of goal attainment can effectively promote mutual goal‐setting and self‐care strategies, enabling individuals with MS to improve their self‐care goals, quality of life and activities of daily living (Payamani et al., 2023).

Individuals with MS experience various symptoms and complications due to the nature of the disease and medication, so nursing theorists emphasize these issues (Hayre‐Kwan et al., 2021; King, 1992). Appropriate solutions to meet basic needs such as nutrition, sleep and rest, elimination, and promotion of physical activity are emphasized. In the present study, the patient and nurse achieved this by formulating mutual goals and using mutual solutions. Our findings showed that collaborative care could provide and interact with many strategies to control MS symptoms and complications. Our participants were able to participate in setting strategies and implementing them according to their disease and abilities. They experienced applying strategies for various complications, such as nutritional behaviours, sleep and rest, constipation relief, promotion of physical activity and exercise, fatigue reduction and stress management. Additional studies are necessary to explore the process of the ‘Exchange of Targeted Behaviors’, a crucial aspect of collaborative care, where both the nurse and patient agree upon specific strategies to manage various aspects of the disease. These studies can inform the development of effective and personalized care plans for individuals with chronic illnesses, ultimately improving their quality of life.

### 4.3 Limitations of the study

While our study is the first to explore the experiences of individuals with MS participating in collaborative nursing care, it has some limitations. The small number of participants in our initial quantitative study resulted in a limited sample size for this qualitative study. As a result, the generalizability of our findings may be limited to only comparable settings. Additionally, the findings may be influenced by cultural and community differences, despite the researchers' efforts to select participants from a diverse range of backgrounds.

## 5 CONCLUSION

As a result of our study, individuals with MS experienced collaborative care as three distinct processes: ‘Beginning of Communication’, ‘Mutual Interaction’ and ‘Mutual Agreement of Care Solutions’. During the ‘Beginning of Communication’, both parties initiated the process by introducing themselves and getting acquainted with each other. This led to the formation of trust, which was an essential factor in the success of the collaborative care process. ‘Mutual Interaction’ was a continuous process of nurse–patient ‘Dialogue’, ‘Mutual Goal Setting’ and ‘Mutual Agreement on Care Solutions’. The ‘Exchange of Targeted Behaviors’ was an important aspect of collaborative care, where both the nurse and patient agreed upon specific strategies to manage various aspects of the disease, including ‘"Nutritional Behaviors’, ‘Sleep and Rest’, ‘Constipation Relief’, ‘Promotion of Physical Activity and Exercise’, ‘Fatigue Reduction’ and ‘Stress Management’.

The findings of our study can be valuable in developing collaborative care programmes for individuals with MS in different settings, as well as for those with other chronic diseases and their families. Further studies are needed to design, implement and evaluate collaborative care programmes for patients with MS and other chronic diseases.

## CONFLICT OF INTEREST STATEMENT

The researchers declare that there was no conflict of interest in the process of implementation, extraction and report of the findings of the present study.

## RESEARCH ETHICS COMMITTEE APPROVAL

The Research Ethics Committee of Hamadan University of Medical Sciences approved the study with the number IR.UMSHA.REC.1399.773.

## Data Availability

Data openly available in a public repository that issues datasets with DOIs
